# Hypersensitivity to antiepileptic drugs in children

**DOI:** 10.1186/2045-7022-4-S3-P144

**Published:** 2014-07-18

**Authors:** Marina Atanaskovic-Markovic, Biljana Medjo, Dimitrije Nikolic

**Affiliations:** 1 University Children's Hospital of Belgrade, Medical Faculti University of Belgrade, Serbia; 2 University Children's Hospital of Belgrade, Medical Faculti University of Belgrade, Intensive Unit, Serbia; 3 University Children's Hospital of Belgrade, Medical Faculti University of Belgrade, Neurology, Serbia

## Beckground

Aromatic (lamotrigin, carbamazepine, phenobarbital) and non aromatic antiepileptic (valproat, topiramate) drugs are frequently associated with hypersensitivity reactions mainly cutaneous such as maculopapular exanthems and bullous and pustular eruptions on the basis of their clinical, cellular and molecular pathophysiology. They usually occur more than 1 hour after the last drug administration and are self-limiting and benign. However, some severe life-threatening reactions have been reported characterized by high fever, malaise, erythema, skin blistering and ulceration of mucous membrans, or hypersensitivity syndromewith fever, lymphadenopaty and systemic symptoms with cutaneous eruption. The underlying mechanisms of these manifestations are not yet completely understood. A cell-mediated pathogenic mechanism has been demonstrated in some cases on the basis of positive patch tests and/or lymphocyte transformation test. The arm of this study was to confirm or rule out the diagnosis of hypersensitivity reactions to antiepileptic drugs in children.

## Method

At University Children's Hospital in Belgrade a group of 62 children with suspected hypersensitivity reactions to antiepileptic drugs were tested for the last 10 years. Out of the total of 62 tested children, 28 were boys and 34 were girls. The ages ranged from 3 years to 17 years (mean age 8.56). Various clinical reactions were described as being induced by drugs (the number of patients affected is shown in parentheses): maculopapular rash (37), maculopapular rash and fever (2) urticaria (14), urticaria and fever (5), urticaria and angioedema (4) The time period that had elapsed from the occurrence of reaction to the performance of patch tests varied from 1 month to one year. Patch tests were performed with culprit drug such as lamotrigine (in 30 children), valproat (in 5 child), carbamazepine (in 24 children), and phenobarbital (in 3 children). In children with a history of mild hypersensitivity reactions and negative patch tests we performed intradermal tests with culprit drugs.

## Results

We found 42 positive patch tests: 18 with lamotrigine, 16 with carbamazepine, 5 with valproat and 3 with phenobarbital. We also found 2 positive intradermal tests only to lamotrigine. None of the 10 control group children displayed positive responses to patch and intradermal tests.

## Conclusion

Our results demonstrate that patch tests are useful for diagnosing anticonvulsant associated cutaneous hypersensitivity reactions, and suggest an cell-mediated pathogenic mechanism.

